# Emerging Orchestrator of Ecological Adaptation: m^6^A Regulation of Post‐Transcriptional Mechanisms

**DOI:** 10.1111/mec.17545

**Published:** 2024-10-05

**Authors:** Ehsan Pashay Ahi, Pooja Singh

**Affiliations:** ^1^ Organismal and Evolutionary Biology Research Programme University of Helsinki Helsinki Finland; ^2^ Institute of Ecology and Evolution University of Bern Bern Switzerland; ^3^ Swiss Federal Institute of Aquatic Science and Technology (EAWAG) Kastanienbaum Switzerland

**Keywords:** adaptation, ecological genetics, molecular evolution, transcriptomics

## Abstract

Genetic mechanisms have been at the forefront of our exploration into the substrate of adaptive evolution and phenotypic diversification. However, genetic variation only accounts for a fraction of phenotypic variation. In the last decade, the significance of RNA modification mechanisms has become more apparent in the context of organismal adaptation to rapidly changing environments. RNA m^6^A methylation, the most abundant form of RNA modification, is emerging as a potentially significant player in various biological processes. Despite its fundamental function to regulate other major post‐transcriptional mechanisms such as microRNA and alternative splicing, its role in ecology and evolution has been understudied. This review highlights the potential importance of m^6^A RNA methylation in ecological adaptation, emphasising the need for further research, especially in natural systems. We focus on how m^6^A not only affects mRNA fate but also influences miRNA‐mediated gene regulation and alternative splicing, potentially contributing to organismal adaptation. The aim of this review is to synthesise key background information to enhance our understanding of m^6^A mechanisms driving species survival in dynamic environments and motivate future research into the dynamics of adaptive RNA methylation.

## 1 Introduction

Fine‐tuning gene expression is crucial for acclimation and adaptation, enabling organisms to rapidly adjust to new environments and selective pressures (Stroud and Losos 2016). Gene expression regulation and genetic responses to environmental changes are related but distinct mechanisms through which organisms interact with their surroundings (Kontarakis and Stainier 2020; López‐Maury, Marguerat, and Bähler 2008; Sztal and Stainier 2020). Gene expression regulation manages immediate cellular processes, controlling gene activation from transcription to post‐translational adjustments, facilitating quick adjustment to both internal and external stimuli. Whereas genetic changes encode long‐term adaptations across generations, driven by natural selection, phenotypic plasticity and genetic assimilation (Ehrenreich and Pfennig 2016), and occasionally epigenetic modifications that affect gene expression without altering DNA sequences (Kontarakis and Stainier 2020; Sztal and Stainier 2020). Studying gene expression regulation in light of genetic responses is crucial for understanding evolutionary adaptation, revealing how immediate cellular reactions and long‐term genetic changes converge to enhance an organism's adaptability and evolutionary success.

Alternative splicing and microRNAs (miRNAs) are two pivotal post‐transcriptional mechanisms that are part of this regulatory process (Keren, Lev‐Maor, and Ast 2010; Singh and Ahi 2022; Stroud and Losos 2016; R. Zhang and Su 2009). By modulating the stability and translation of mRNAs, miRNAs enable organisms to rapidly reconfigure gene regulatory networks in response to environmental signals, improving their survival and reproductive success (Chiou 2007; Song et al. 2019; Xu et al. 2019). Alternative splicing can generate different proteins with distinct functions by selectively including or excluding exons (or introns) in mRNA transcripts (Keren, Lev‐Maor, and Ast 2010). This diversification of the functional repertoire of the genome can allow organisms to adapt to various ecological niches and challenges (reviewed in Singh and Ahi 2022) and contribute to ecologically diverse adaptive radiations (Duenser et al. 2024; Singh et al. 2021). The precision and adaptability afforded by miRNAs and alternative splicing are essential for organisms to optimise their gene expression and thrive in dynamic landscapes of evolutionary change.

**FIGURE 1:**
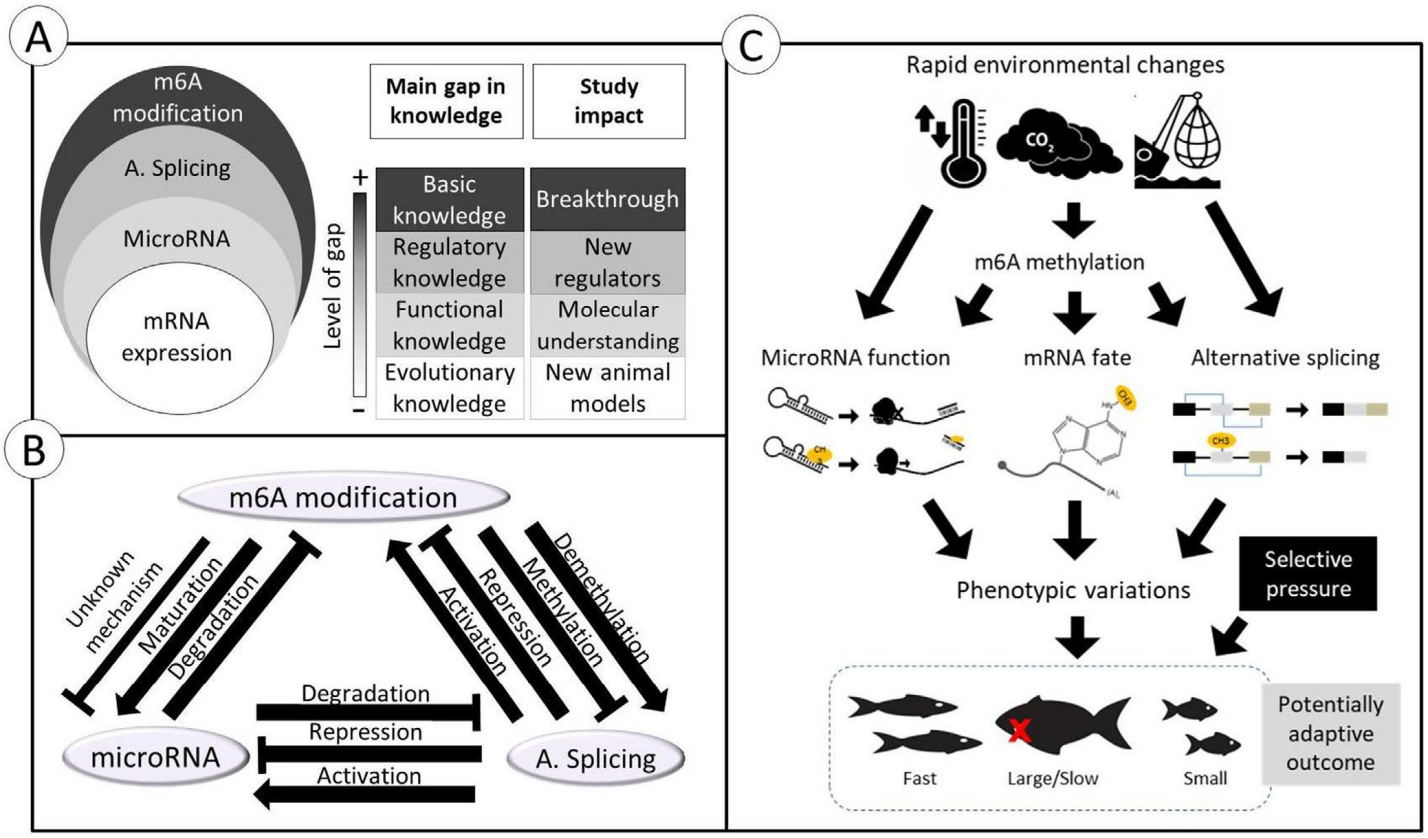
m^6^A RNA methylation in the context of environmental acclimation/adaptation. (A) This panel presents the current understanding of (post‐) transcriptional regulations in ecological adaptation. Darker shades indicate areas with less knowledge about the role of each mechanism. The first column highlights the primary knowledge gaps of each mechanism, marked by the corresponding shade, while the second column outlines potential outcomes if further studies are pursued in these areas. (B) This panel depicts a three‐way model of known regulatory interactions between m^6^A RNA modification, miRNA and alternative splicing. These reciprocal and interconnected regulatory interactions represent a variety of regulatory outcomes at the post‐transcriptional level, which could lead to a broad spectrum of molecular responses when each mechanism is differently influenced by external and internal stimuli. The arrows and blockheads indicate various stimulatory and inhibitory post‐transcriptional crosstalk between these processes. (C) This panel illustrates an example of how m^6^A RNA methylation contributes directly or indirectly to advantageous phenotypic variation in response to rapid environmental change—either through directly affecting mRNA fate or indirectly through the regulation of two major post‐transcriptional regulatory mechanisms (miRNA and alternative splicing) when exposed to environmental stimuli.

N6‐methyladenosine (m^6^A) modification of RNA is another pivotal post‐transcriptional regulatory mechanism (Yue, Liu, and He 2015). While DNA methylation has been studied in the context of acclimation, adaptation and speciation (Ashe, Colot, and Oldroyd 2021), the role of m^6^A RNA modifications remains largely unexplored (Hu et al. 2022; Ma et al. 2017) (Figure 1A). This chemical modification is known for its dynamic and reversible nature and thus has the potential to influence gene expression, mRNA stability, alternative splicing (Adhikari et al. 2016; Bartosovic et al. 2017) and miRNA‐mediated regulation (Feng et al. 2023; Mei et al. 2023). Given its conservation across species and its capacity to rapidly alter gene expression profiles (Liu et al. 2022; Zhao et al. 2021), m^6^A has emerged as a significant player in the fine‐tuning of genetic responses to changing environmental conditions (Hu et al. 2022).

In this review article, we synthesise what is known about the role of m^6^A RNA methylation in the context of adaptive responses of organisms to environmental changes (which may also have evolutionary outcomes). We emphasise the need for further exploration in this field, particularly in natural systems (see the knowledge gap in Figure 1A). Our primary focus is to highlight the role of m^6^A as an adaptive orchestrator, which extends beyond its direct influence on mRNA fate, to its regulation of two prominent post‐transcriptional regulatory mechanisms: miRNA‐mediated gene regulation and alternative splicing (Figure 1B). By doing so, we underscore the ways in which m^6^A might contribute to rapid acclimation and potentially adaptation of organisms. This will help us better understand the dynamic mechanisms that underlie the diversification and survival of species in constantly changing environments (Figure 1C). Throughout this review, the terms ‘adaptive responses’ and ‘adaptation’ primarily refer to rapid and short‐term responses that may enhance the adaptive capabilities of an individual or population. We also discuss how ecologically important m^6^A RNA modifications may stem from genetic changes, such as mutations in genes associated with the m^6^A RNA modification process (e.g., m^6^A erasers, writers, readers and their regulators and partners). Such m^6^A‐mediated ecological and environmental responses may lead to long‐term adaptation through natural selection and be governed by heritable genetic variation in m^6^A modifiers.

## 2 The Emerging Roles of N6‐Methyladenosine (m^6^A) in Adaptation

The emerging field of epitranscriptomics, the study of chemical modifications to RNA molecules, has shed new light on the potential role of N6‐methyladenosine (m^6^A) in evolutionary adaptation (Antoine et al. 2021; Dannfald, Favory, and Deragon 2021; Miao et al. 2022). m^6^A is one of the most prevalent and dynamic RNA modifications found in eukaryotic organisms (Jiang et al. 2021). These modifications are well known for their regulatory functions in mRNA stability, splicing and translation. m^6^A modifications were found to be evolutionarily conserved over 500 Mya of plant evolution (Miao et al. 2022) and 30 Mya of primate evolution (Ma et al. 2017), illustrating that they are evolving under selective constrains. Recent research has highlighted the significance of m^6^A in myriad biological processes that may lead to environmental acclimation/adaptation (Miao et al. 2022; Zhang et al. 2020). The ability of m^6^A modifications to rapidly modulate gene expression to environmental change makes this mechanism crucial for the survival of organisms facing environmental flux. For example, m^6^A modifications have been implicated in the regulation of stress responses (Yang et al. 2022), immune system function (Zhang, Fu, and Zhou 2019) and developmental processes (Shao et al. 2021), all of which are crucial for an organism to adjust to its habitat. m^6^A modifications are potential regulators of rapid adaptive response during exposure to environmental chemicals (Li et al. 2020). In insects, for instance, m^6^A‐dependent changes in mRNA fate of metabolic genes can contribute to developing rapid resistance to insecticides (Muthu Lakshmi Bavithra et al. 2023). In plants, m^6^A‐dependent changes in various RNA targets (mRNA of senescence‐related genes and miRNAs) are implicated in regulating response to early senescence and adaptation to darkness (Sheikh et al. 2024). In apple trees, it was recently found that an m^6^A writer (MdMTA) mediates drought acclimation by promoting the mRNA stability and translation efficiency of genes involved in lignin deposition and scavenging reactive oxygen species (Hou et al. 2022). Such examples make it conceivable that some of these m^6^A‐mediated regulatory changes in response to environmental stressors significantly contribute to rapid adjustment across the tree of life. Climate change will expose organisms to environmental flux—understanding if (and how) dynamic m^6^A modifications play a role in climate adaptation is an unexplored and pressing line of enquiry.

m^6^A has been linked to the evolution of complex traits and phenotypes. It can not only affect individual gene expression but also alter regulatory networks governing entire biological processes (An et al. 2020; Chang et al. 2017). A recent study in ants found that m^6^A modification regulates mRNA stability of genes with pivotal rule in orchestrating complex behavioural and physiological traits required for the division between foragers and nurses (Chen et al. 2024). This discovery demonstrates for the first time that complex traits can be tightly regulated by an environmentally responsive post‐transcriptional mechanism such as m^6^A modification in social insects (Chen et al. 2024). Another example of complex adaptive responses to environmental stress was reported in the evolution of heat shock protein expression (Chen, Feder, and Kang 2018), in which m^6^A modification of heat shock mRNAs (e.g., by affecting their stability and translation) was one of the contributing factors to adaptive heat stress response (Zhou et al. 2015). These examples also suggest that m^6^A modifications may play an important role in the evolution of novel traits and adaptive strategies in species. The evolutionary conservation of m^6^A‐related proteins across diverse species underscores the importance of this modification in wide range of evolutionary conserved cellular process (Liu et al. 2022). These proteins are present in organisms ranging from yeast to humans, implying that m^6^A has played a fundamental role in the evolution of life on earth.

Although the inheritance of m^6^A RNA modifications is not yet well studied, they may have transgenerational effects through several mechanisms. For example, maternally inherited m^6^A‐modified RNAs could influence early developmental processes in offspring by affecting mRNA stability and translation, potentially altering gene expression during development (Zhu et al. 2023). Another study demonstrated that genetic variants associated with m^6^A levels in mRNA transcripts, known as m^6^A‐QTLs, contribute to the heritability of various immune and blood‐related traits (Zhang et al. 2020). Most of these m^6^A‐QTLs were found to be located in the binding sites of various regulators, including miRNAs and transcription factors (Zhang et al. 2020). The enzymes and components that regulate m^6^A modifications, that is, writers, erasers and readers, are encoded by inheritable genes, which mean genetic variations in these enzymes can influence m^6^A patterns across generations. Emerging evidence also suggests that RNA molecules, including those with m^6^A modifications, might be transferred to offspring under specific conditions, such as via sperm, possibly affecting early embryonic gene expression (Gui and Yuan 2021; Xiong et al. 2024; Zhang et al. 2023). This highlights the potential for m^6^A to influence heritability, although more research is needed to fully understand these mechanisms.

Taken together, the emerging role of N6‐methyladenosine (m^6^A) in ecological acclimation/adaptation represents an exciting frontier in evolutionary biology. By modulating gene expression in response to changing environments and potentially influencing the development of complex traits, m^6^A modifications may influence evolutionary processes. Further research in this field promises to unveil the ways in which epitranscriptomic modifications may contribute to the process of evolutionary adaptation.

## 3 Alternative Splicing and miRNAs: Two Major Protagonists Exerting the Effects of m^6^A Modification

N6‐methyladenosine (m^6^A) has emerged as a central player in the intricate web of post‐transcriptional gene regulation. m^6^A can exert its influence upstream of two major processes: microRNA (miRNA) biogenesis (Erson‐Bensan and Begik 2017) and alternative splicing (Covelo‐Molares, Bartosovic, and Vanacova 2018). This dual regulatory role of m^6^A highlights both its versatility in shaping gene expression and its significance in cellular and organismal physiology.

First, m^6^A regulates miRNA functions and biogenesis at various stages (Erson‐Bensan and Begik 2017; Han, Guo, and Fan 2021). miRNAs, small non‐coding RNAs, function as post‐transcriptional regulators, allowing for the rapid adjustment of gene expression (Loh, Yi, and Streelman 2011). Their ability to target and modulate multiple mRNAs provides a versatile mechanism for organisms to adjust to new challenges (Berezikov 2011). Hypothetically, during acclimation, miRNAs can help fine‐tune the expression of genes involved in processes like immune response, stress tolerance and developmental changes, thus aiding in an organism's ability to thrive in dynamic environments. Furthermore, miRNAs can evolve rapidly, generating new miRNA species and expanding regulatory networks, which can contribute to species‐specific adaptations (Loh, Yi, and Streelman 2011). Over the past decade, growing evidence also confirmed that miRNAs can play significant role in adaptation to different environments through mechanisms involving variation in their expression in response to a wide variety of external factors and stressors (Barrio, Dekanty, and Milán 2014; Chen, Li, and Xiong 2012; Gajigan and Conaco 2017; Kelley et al. 2021; Torma et al. 2020), functional innovation via newly evolved miRNAs (Graham and Barreto 2019; Mohammed et al. 2014; Sun et al. 2022), functional ramifications during development which may ultimately lead to organismal fitness (Bianchi et al. 2017; Kosik and Nowakowski 2018; Malnou et al. 2019; Roberts et al. 2024; Seistrup et al. 2023) and contribute to adaptive radiation by increasing the diversity of transcriptional responses in relatively short period of time (Franchini et al. 2016, 2019). A critical stage in miRNA biogenesis, known as maturation, involves the processing of pri‐miRNAs by the enzymes Drosha and Dicer (Loh, Yi, and Streelman 2011). This process first converts pri‐miRNAs (primary miRNAs) into pre‐miRNAs (precursor miRNAs) as an intermediate step, and then into mature, double‐stranded miRNAs that regulate gene expression by targeting specific mRNA sequences for degradation or translational repression. The m^6^A modification can be strategically placed within pri‐miRNAs and pre‐miRNAs, influencing their processing by the microprocessor complex and affecting the efficiency of miRNA maturation (Han, Guo, and Fan 2021). Moreover, m^6^A modifications within the 3′ untranslated regions (UTRs) of target mRNAs can influence miRNA‐mediated post‐transcriptional regulation by modulating miRNA–mRNA interactions (Erson‐Bensan and Begik 2017). This regulatory interplay enables m^6^A to regulate miRNA expression levels and their target specificity, which in turn affects the overall post‐transcriptional gene regulatory landscape.

Second, m^6^A modifications also play a crucial role in alternative splicing. The recognition of splice sites by spliceosomal complexes or splicing regulatory proteins can be influenced by m^6^A within pre‐mRNA sequence (Bartosovic et al. 2017; Adhikari et al. 2016). The selection of specific exons or splice variants is therefore indirectly under the control of the m^6^A modifications that can modulate the functional properties of the resulting proteins. By acting upstream of alternative splicing, m^6^A provides a mechanism to rapidly reprogram gene expression profiles in response to changing environmental conditions or developmental cues, ultimately contributing to an organism's adaptability and evolutionary fitness. Hence, the ability of m^6^A to control both miRNA functions and alternative splicing underscores the multifaceted role of m^6^A in gene expression regulation, positioning it as a central player in the complex orchestra of cellular and molecular biology with potentials for driving adaptive responses through the post‐transcriptional mechanisms (Figure 1B,C). Alternative splicing is a key player in the realm of adaptive evolution plants and animals (Duenser et al. 2024, 2023; Jacobs and Elmer 2021; Keren, Lev‐Maor, and Ast 2010; Lecaudey et al. 2021, 2019; Price et al. 2018; Salisbury, Delgado, and Dalziel 2021; Singh et al. 2017; Smith et al. 2018). Splicing acts as a substrate for adaptation by enhancing the coding potential of the genome from standing or de novo genetic variation (Singh and Ahi 2022). Through alternative splicing, organisms can generate new protein variants or adjust the expression of existing ones, depending on the environmental context (Keren, Lev‐Maor, and Ast 2010). This plasticity is particularly important in complex organisms, such as vertebrates, where alternative splicing contributes to tissue specialisation and developmental flexibility (Baralle and Giudice 2017). By providing a rich source of transcriptional and proteomic variation, alternative splicing enables organisms to adapt to novel ecological niches and evolving challenges, thereby promoting their long‐term survival (Shang, Cao, and Ma 2017) and phenotypic diversification (Wright, Smith, and Jiggins 2022).

Finally, it is also important to recognise the extensive functional connections between miRNAs and alternative splicing. These mechanisms do not operate in isolation; instead, they collaborate intricately to fine‐tune gene expression. miRNAs can target and modulate the expression of splicing factors, thereby influencing the selection of specific splice variants. Conversely, alternatively spliced transcripts can encode miRNA binding sites, allowing them to serve as regulatory targets (Ratnadiwakara, Mohenska, and Änkö 2018). This crosstalk between miRNAs and alternative splicing provides a dynamic and likely more malleable and adaptable layer to gene regulation. It enables organisms to respond rapidly to environmental changes and evolutionary pressures by not only adjusting the quantity of gene products but also by diversifying their functional properties, ultimately facilitating ecological acclimation and adaptation. The complexity of regulatory interactions between m^6^A, miRNA and splicing that makes them so relevant for adaptive evolution also makes it challenging to study these processes.

## 4 Scenarios Linking m^6^A Components to Alternative Splicing and miRNAs

**FIGURE 2:**
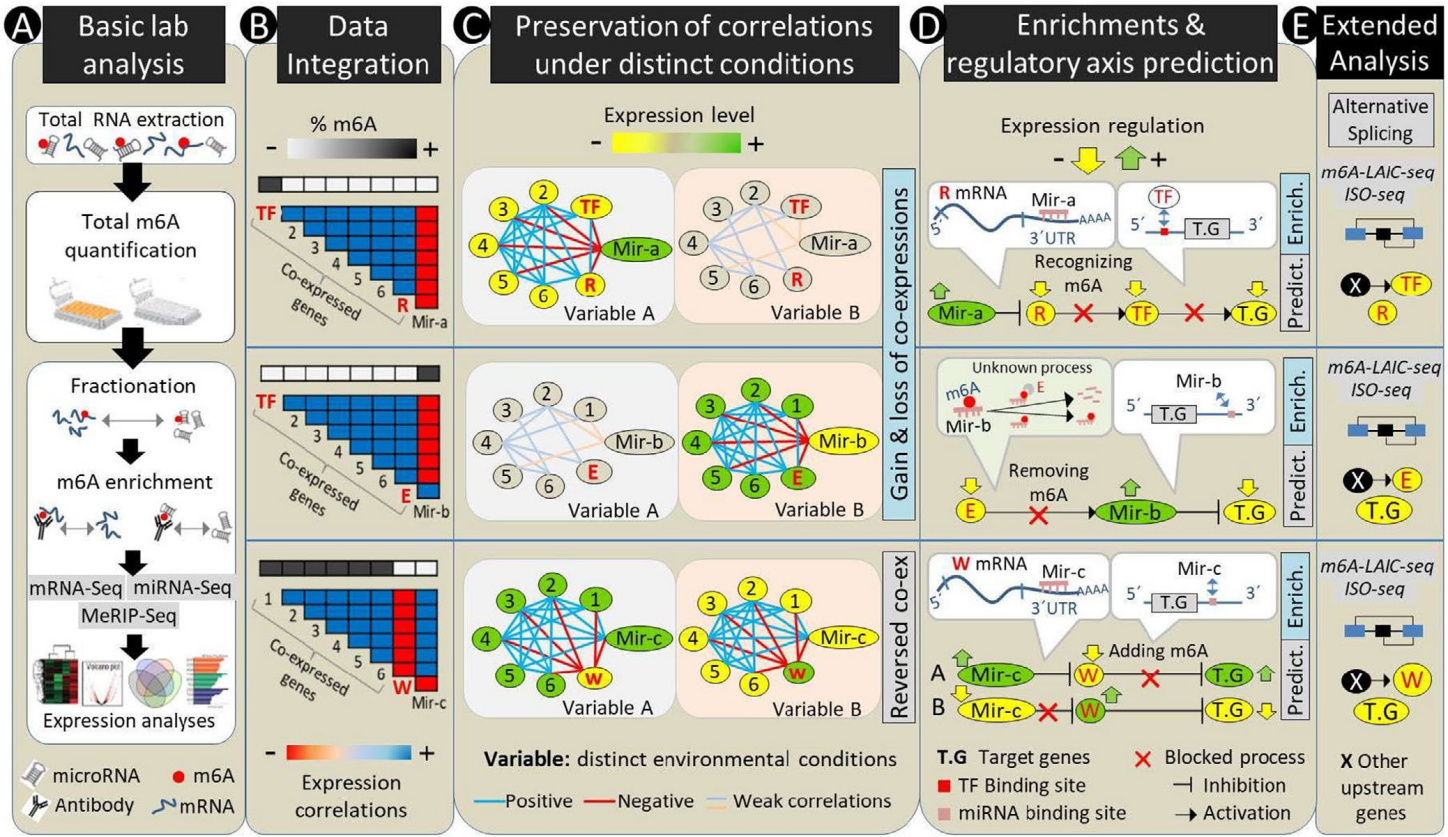
An example representing a three‐way investigation of m^6^A‐mediated regulation of transcriptional responses. (A) Standard laboratory methods and analyses to identify differences in mRNA, miRNA and m^6^A‐methylated RNAs (i.e., m^6^A–mRNA and m^6^A–miRNA) using the same biological samples. Conventional RNAseq methods can be used for all these quantifications; however, an immunopercipitation step is incorporated for the molecular enrichment of the m^6^A‐methylated RNAs prior to their sequencing (MeRIP‐seq method). (B) Integration of expression data from all the methods through combination of various correlation analyses (i.e., correlation between expression of miRNAs and mRNAs as well as their correlation with m^6^A levels on the same mRNA/miRNA transcripts). (C) Investigation of differences in correlations between all molecular components under distinct environmental conditions (e.g., to check if the correlations are present or absent under different conditions). (D) Prediction of regulatory axis between m^6^A methylation markers, miRNAs and target genes (e.g., through various binding site enrichment analyses of genes identified within each of the co‐expression module from the previous step). (E) Extension of the predicted axis through identification of differentially expressed isoforms of the identified regulators or their potential upstream effectors. The abbreviations R, W, E, TF and Mir‐(A, B, C) stand for reader, writer, eraser, transcription factor and microRNA‐(A, B, C), respectively.

The effects of N6‐methyladenosine (m^6^A) modification on miRNA biogenesis, functions and alternative splicing are highly nuanced and context‐dependent, primarily determined by the interplay of its different molecular components: writers, erasers and readers (Zaccara, Ries, and Jaffrey 2019). Depending on the specific cellular and environmental context, the balance and interplay of these components can result in a wide spectrum of regulatory outcomes, from fine‐tuning miRNA‐mediated gene regulation to shaping the diversity of mRNA isoforms through alternative splicing (Figure 2). This complex regulatory network highlights the multifaceted nature of m^6^A modification in post‐transcriptional gene regulation and its potential in adaptation.

### 4.1 M^6^A Writers

Within the context of potential adaptive evolution, the role of m^6^A writers, specifically the methyltransferase complex led by METTL3, is multidimensional and instrumental in orchestrating regulatory processes such as miRNA biogenesis and alternative splicing. These writers deposit m^6^A marks on RNA transcripts, and depending on their distribution, they effect critical aspects of post‐transcriptional gene regulation (Feng et al. 2023). In the realm of miRNA biogenesis, m^6^A modifications can be strategically placed within pri‐miRNA and pre‐miRNA sequences. These modifications can influence the processing of these primary miRNA transcripts by the microprocessor complex, consisting of Drosha and DGCR8. Drosha is a ribonuclease III enzyme that, along with its cofactor DGCR8 (DiGeorge Syndrome Critical Region 8), forms the microprocessor complex responsible for the initial processing of pri‐miRNAs into pre‐miRNAs in the nucleus. This can enhance or inhibit Drosha cleavage, thus modulating the efficiency of miRNA maturation (Su et al. 2023). In an adaptive evolutionary context, this regulatory capability can be particularly relevant, as it may allow organisms to fine‐tune the production of specific miRNAs in response to environmental changes or developmental cues. In plants, for instance, changes in miRNA biogenesis (through altering pri‐miRNA accumulation and processing) have been shown to play crucial role during development and adaptation to environmental changes (Zhang, Liu, and Yu 2015). Thus, the precise m^6^A‐dependent control over miRNA biogenesis can be a probable mechanism enabling the rapid response of miRNA‐mediated gene regulation networks, which may further contribute to the optimisation of an organism's fitness and survival in ever‐changing ecological niches. For instance, m^6^A‐dependent changes in miRNA biogenesis have been implicated in regulating gene expression during environmental exposures to toxicants (Li et al. 2020). In plant, m^6^A‐dependent changes in miRNA biogenesis, through activation of MTA; an m^6^A writer homologous to METTL3 in animals, are implicated in regulating the biological response to early senescence, potentially contributing to adjustment to changing environments (Bhat et al. 2020; Sheikh et al. 2024). The regulatory crosstalk between miRNA and m^6^A modification is reciprocal. A study in rats showed that stressful conditions can stimulate the expression of miR‐124‐3p, which regulates the fat mass‐ and obesity‐associated (FTO) enzyme, thereby affecting FTO‐dependent m^6^A methylation of neural plasticity gene mRNAs in the hippocampus (Roy, Ochi, and Dwivedi 2022). These changes can lead to a range of adaptive and maladaptive behavioural outcomes. However, it is still uncertain whether such varied brain responses to environmental stressors in nature will lead to adaptive behavioural changes after prolonged exposure.

m^6^A writers can exert their influence on alternative splicing by the deposition of m^6^A marks on pre‐mRNA transcripts can impact the recognition of splice sites by the spliceosomal complexes or splicing regulatory proteins (Mendel et al. 2021). Depending on the location and density of m^6^A modifications, this can promote or hinder the inclusion or exclusion of specific exons, leading to the generation of alternative mRNA isoforms with distinct functional properties (Adhikari et al. 2016). In an adaptive evolutionary context, this means that m^6^A can facilitate the rapid reprogramming of gene expression profiles, allowing organisms to swiftly adjust to novel environmental conditions or selective pressures by altering the functional properties of the proteins produced. This dynamic regulation of alternative splicing, influenced by m^6^A writers, can potentially contribute to the genomic plasticity and diversification necessary for evolutionary adaptation. In honey bees (*Apis mellifera*), extensive and simultaneous m^6^A RNA modification and alternative splicing events have been identified as underlying mechanisms differentiating the development of worker and queen castes, despite having identical female genomes (He et al. 2022; Wang et al. 2021). Although the detailed regulatory crosstalk between m^6^A RNA modification and alternative splicing during this process remains unclear, the involvement of three m^6^A writers at different developmental stages (AmMETTL3, AmMETTL14 and AmWTAP) is well characterised (Wang et al. 2021).

### 4.2 M^6^A Erasers

The role of m^6^A erasers, specifically demethylases like FTO and ALKBH5, is critical in shaping microRNA (miRNA) biogenesis, functions and alternative splicing dynamics (Chen et al. 2021; Erson‐Bensan and Begik 2017; Feng et al. 2023). These enzymes function to remove m^6^A modifications from RNA transcripts, allowing for dynamic and reversible regulation of post‐transcriptional processes. In the realm of miRNA biogenesis, m^6^A erasers can influence this process by demethylating pri‐miRNAs and pre‐miRNAs, potentially affecting their processing by the microprocessor complex (Drosha and DGCR8) (Su et al. 2023). The demethylation of m^6^A marks in these regions can alter the cleavage efficiency of Drosha, leading to changes in miRNA maturation. In the context of adaptive evolution, this dynamic regulation can allow organisms to rapidly adjust the production of specific miRNAs in response to shifting environmental conditions or developmental cues. The ability to fine‐tune miRNA biogenesis through m^6^A demethylation enables a swift and flexible response to evolutionary challenges, optimising miRNA‐mediated gene regulation networks and enhancing an organism's fitness and adaptability.

m^6^A erasers play a pivotal role in modulating alternative splicing through the removal of m^6^A marks from pre‐mRNA transcripts can affect splice site recognition by the spliceosomal complexes or splicing regulatory proteins (Bartosovic et al. 2017). Depending on the precise location and density of m^6^A demethylation, this can either promote or inhibit the inclusion or exclusion of specific exons, ultimately generating alternative mRNA isoforms with distinct functional properties. For instance, in FTO knockout mice, exons that are normally excluded in wild‐type conditions were included. This is attributed to the absence of FTO, which increases m^6^A levels around splice sites and enhances the RNA‐binding ability of the SRSF2 protein (an exonic splicing enhancer) in these regions (Zhao et al. 2014). Conversely, although to a lesser extent, m^6^A demethylation might promote the inclusion of typically excluded exons by reducing the binding of repressive splicing factors, potentially allowing for the inclusion of previously suppressed exons (Bartosovic et al. 2017). While evidence for this inclusion scenario is less substantial than for exon exclusion, the overarching influence of m^6^A on splicing suggests that its removal could logically lead to shifts towards exon inclusion in specific contexts. Thus, m^6^A erasers may allow organisms to rapidly reprogram gene expression profiles, responding quickly to novel environmental conditions or selective pressures by modifying the functional properties of the resulting proteins. This dynamic regulation of alternative splicing, influenced by m^6^A erasers (Zhu et al. 2023), might also contribute to genomic plasticity and the diversification necessary for adaptation to changing environments.

### 4.3 M^6^A Readers

m^6^A readers encompass a diverse range of RNA‐binding proteins that have emerged as key players in regulating miRNA biogenesis, functions and alternative splicing (Adhikari et al. 2016; Feng et al. 2023; Zhu et al. 2023). These readers recognise and interpret m^6^A modifications on RNA transcripts, and their diversity allows for highly nuanced and context‐dependent regulation. In miRNA biogenesis, m^6^A readers can influence the processing of pri‐miRNAs and pre‐miRNAs by binding to m^6^A‐modified regions (Su et al. 2023). Some m^6^A readers may enhance the recruitment of the microprocessor complex (Drosha and DGCR8) to pri‐miRNAs by recognising the m^6^A marks, thereby facilitating efficient miRNA maturation. Other m^6^A readers may compete with miRNA processing factors, limiting the accessibility of the microprocessor complex to m^6^A‐modified sites and suppressing miRNA biogenesis (Su et al. 2023). The interplay of m^6^A readers can hypothetically fine‐tune the production of specific miRNAs in response to environmental changes. This topic is still in its infancy, and to date, no study has investigated whether m^6^A reader‐mediated effects on miRNA biogenesis in response to environmental stimuli can translate into advantageous results at cellular or organismal levels. For instance, exposure to inhaled toxins has been shown to excessively promote the m^6^A‐mediated maturation of miR‐25, a process facilitated by an m^6^A reader, NKAP, which in turn provokes malignant phenotypes in cells (Zhang et al. 2019). However, it remains unknown whether genetic variations in the NKAP reader that reduce its activity as an m^6^A reader can enhance cellular resistance and thereby organismal adaptation to these toxins.

Regarding alternative splicing, m^6^A readers play a key role in modulating splice site recognition and the splicing regulatory landscape (Adhikari et al. 2016). These readers can bind to m^6^A‐modified regions within pre‐mRNA transcripts, influencing the recruitment of splicing factors or spliceosomal complexes (Adhikari et al. 2016). Depending on the identity and function of the specific m^6^A reader involved, this interaction can either enhance or inhibit exon inclusion or exclusion, leading to the generation of alternative mRNA isoforms with distinct functional properties. The remarkable diversity of m^6^A readers allows for a wide range of possible outcomes in alternative splicing regulation, and this diversity can be a potent driver of adaptive responses (Zhen et al. 2020). In honey bees, the extensive m^6^A RNA modification and alternative splicing events that shape the distinct development of worker and queen castes (He et al. 2022; Wang et al. 2021) also involve two major m^6^A readers; AmYTHDC1 and AmYTHDF (Wang et al. 2021). While further studies are needed to fully understand the roles of these readers in the interaction between m^6^A modifications and alternative splicing, it is already established that YTHDC1 directly regulates alternative splicing by recruiting and modulating pre‐mRNA splicing factors, thus facilitating their access to the binding regions of targeted mRNAs (Xiao et al. 2016). By providing multiple avenues for regulatory fine‐tuning, m^6^A readers may potentially contribute to the adaptability of organisms, allowing them to rapidly reprogram gene expression profiles to respond to evolving environmental conditions or selective pressures. In this way, m^6^A readers add another layer of complexity to the multifaceted effects of m^6^A modifications in post‐transcriptional gene regulation, acclimation and potentially adaptive evolution (Box 1).

BOX 1 **Evolution of m^6^A and Its Similarity and Differences to microRNA and AS Evolution.**

m^6^A modifications demonstrate significant evolutionary conservation across a wide range of species, from single‐celled microorganisms to complex multicellular organisms like humans (Zaccara, Ries, and Jaffrey 2019). The evolutionary significance of m^6^A may lie in its ability to provide rapid adaptive advantages by dynamically regulating gene expression and RNA processing (Hu et al. 2022; Lei et al. 2023; Li et al. 2020). These adjustments not only enhance organismal survival but may also contribute to the evolution of novel traits and functions. m^6^A modifications, microRNAs and alternative splicing, all contribute to the complex landscape of post‐transcriptional gene regulation, each with distinct evolutionary paths. These processes have evolved in response to selective pressures and developmental demands, shaping the diversity and complexity of gene regulation across the tree of life (Loh, Yi, and Streelman 2011). Comparing the evolution of m^6^A, microRNA and alternative splicing provides valuable insights into how RNA‐related processes have evolved over time.

**Similarities:**

***Conservation of Core Components*:** A commonality among m^6^A, microRNA and alternative splicing is the evolutionary conservation of their core machinery, that is, key proteins orchestrating these processes across species (Ast 2004; Ma et al. 2017; Merkin et al. 2012; Miao et al. 2022; Moran et al. 2017; Zaccara, Ries, and Jaffrey 2019).

***Diverse Regulatory Functions*:** All three mechanisms add complexity to gene expression regulation, enabling organisms to adapt to changing environments and developmental stages beyond the genomic code (Liu et al. 2008; Liu and Zhou 2021; Nainar et al. 2016; Yang, Xue, and An 2007).

***Evolutionary Innovation*:** These processes have been fine‐tuned and expanded to meet the specific needs of different organisms, with novel microRNAs, alternative splicing events and m^6^A modification sites emerging to contribute to species‐specific traits and ecological adaptations (Bush et al. 2017; Loh, Yi, and Streelman 2011; Singh et al. 2017).

**Differences:**

***Molecular mechanisms*:** While all three processes involve RNA, they operate differently: m^6^A chemically modifies adenine bases in RNA (Boulias and Greer 2022); microRNAs regulate gene expression by binding to mRNAs; and alternative splicing rearranges exons and introns to produce different mRNA isoforms.

***Targets and outcomes*:** The targets and outcomes of these processes differ: m^6^A primarily affects mRNA stability, translation and splicing (Boulias and Greer 2022); microRNAs target mRNA for degradation or translational repression; and alternative splicing generates diverse protein isoforms with different functions.

***Evolutionary rates*:** The evolutionary rates of miRNA, alternative splicing and m^6^A RNA modifications vary due to their distinct roles in gene regulation and adaptation (Simkin et al. 2020; Wright, Smith, and Jiggins 2022). While miRNAs are generally conserved, especially those crucial for basic functions, newer miRNAs can evolve rapidly, aiding species‐specific adaptations (Cui, You, and Chen 2017; Loh, Yi, and Streelman 2011; Nozawa, Miura, and Nei 2010; Santpere et al. 2016). Alternative splicing shows variable rates, with essential events being conserved, while other events adapt more flexibly, influencing species‐specific traits (Duenser et al. 2024; Gelfman et al. 2012; Keren, Lev‐Maor, and Ast 2010; Singh et al. 2017). The core machinery for m^6^A RNA modifications remains conserved, but specific m^6^A‐binding sites are largely unconserved, allowing adjustments to environmental changes (Liu, Zhang, and Yeager 2018; Miao et al. 2020; Wong and Eirin‐Lopez 2021).

***Reversibility*:** Reversibility distinguishes m^6^A RNA methylation from miRNA regulation and alternative splicing, which are typically stable and irreversible (Bi et al. 2019). m^6^A modifications offer a dynamic layer of regulation, with marks that can be added, removed and reinstated in RNA transcripts, allowing for rapid adjustment of gene expression to changing environmental cues or selective pressures.

## 5 m^6^A RNA Methylation a Missing Piece of Puzzle in Rapidly Linking Environment to Other Post‐Transcriptional Regulatory Mechanisms

m^6^A RNA modification is recognised as a post‐transcriptional mechanism that influences RNA metabolism and function, although it operates differently from traditional DNA‐based epigenetic modifications such as DNA methylation and histone changes. While m^6^A does not alter the DNA sequence, it can indirectly influence gene expression patterns across cell generations (Fu et al. 2014). Generally, m^6^A modifications that reduce RNA stability do not transmit during mitosis, while those that enhance RNA stability can be transmitted and persist across multiple cell divisions (Boulias and Greer 2022; Kretschmer and Gapp 2022; Xiong et al. 2024; Zhang et al. 2023). Additionally, m^6^A‐related enzymes (writers, readers and erasers) are transmitted during mitosis, allowing them to re‐establish m^6^A modifications in daughter cells, maintaining cellular memory and identity (Bai et al. 2023). The role of m^6^A in meiosis and its potential for intergenerational inheritance is less clear and remains an area of active research. Unlike stable DNA modifications, the transient nature of RNA suggests that m^6^A modifications are less likely to be directly inherited through meiosis, but they may influence early embryonic development and gene regulation in progeny. For instance, sperm RNA profiles (including m^6^A‐methylated RNAs) are easily affected by environmental exposure and can even be inherited for several generations (Gui and Yuan 2021). Interestingly, a new study has revealed that a diet‐induced multigenerational paternal obesity enhances the susceptibility of the offspring to spermatogenesis disorders by increasing METTL3‐mediated m^6^A modification of mRNA of a gene regulating retinoic acid pathway (Xiong et al. 2024). Another study in mouse demonstrated that m^6^A‐mediated increased mRNA stability of a gene regulating cellular senescence during spermatogenesis later affects offspring cognitive ability (Zhang et al. 2023). This process was also strongly affected by paternal exposure to environmental cadmium (Cd) and high‐fat diet (Zhang et al. 2023).

Despite the aforementioned gaps in our understanding, m^6^A RNA methylation has emerged as a potential link between environmental changes and adaptive environmental responses in organisms (Hu et al. 2022; Lei et al. 2023; Li et al. 2020). Its significance lies in its ability to serve as a rapid and dynamic sensor, translating environmental cues into precise post‐transcriptional regulatory adjustments (see examples below). Unlike changes in DNA sequence, which occur over longer timescales, m^6^A modifications can be rapidly added or removed in response to varying environmental conditions. This unique attribute of m^6^A may allow organisms to fine‐tune gene expression profiles even within their lifetime, facilitating their adjustment to a dynamic world. For instance, m^6^A RNA methylation has already been proposed as the reversible pathway evolved to regulate processes that involve rapid expression changes of large groups of genes and proteins (Fu et al. 2014). In an adaptive context, an interesting example of this can be the essential role of m^6^A RNA modification in rapid cellular acclimation to hypoxic conditions (Wang et al. 2021). In response to hypoxia in mammalian cells, the total m^6^A methylation is reduced by upregulation of an m^6^A eraser (ALKBH5) and downregulation of an m^6^A reader (YTHDF2) which promotes mRNA decay (Wang et al. 2021). At the organismal level, protein levels of m^6^A writers increase while readers and erasers are suppressed under extreme cold stress adaptation in wood frogs (Rehman et al. 2023). Although this suggests a role for m^6^A RNA methylation in surviving freezing and regulating a hypometabolic state, the detailed mechanism remains unclear (Rehman et al. 2023). In *Arabidopsis*, upon agriculturally relevant salt treatment, m^6^A is dynamically deposited on and stabilises mRNA encoding proteins required for salt and osmotic stress response, and thereby promoting acclimation to high salt levels (Anderson et al. 2018). A later study demonstrated that this m^6^A‐dependent salt tolerance also involves reducing the mRNA stability of salt stress regulators that have negative effects on cellular homeostasis (Hu et al. 2022). However, only recently has the mechanism underlying this rapid m^6^A‐dependent adaptive response been elucidated, and it involves the rapid induction of *FIONA1* (*FIO1*), a novel m^6^A writer, just a few hours after exposure to high levels of salt (Cai et al. 2024).

m^6^A methylation also serves as a bridge connecting the environment to other major post‐transcriptional mechanisms, particularly miRNA regulation and alternative splicing. In times of environmental stress or adaptation, alterations in miRNA profiles mediated by m^6^A modifications can swiftly reprogram gene regulatory networks to optimise an organism's response. Similarly, m^6^A marks on pre‐mRNA transcripts can modulate alternative splicing events, generating multiple mRNA isoforms with distinct functions. This dynamic regulation may ensure that gene expression can rapidly adjust to changing environmental conditions and potentially allow organisms to adapt to novel ecological niches. For instance, in response to hypoxic stress, an m^6^A reader (YTHDC1) involved in the regulation of alternative splicing is rapidly induced (Wang et al. 2021). This induction leads to increased activity in alternative splicing, resulting in the generation of a diverse array of proteins, as well as enhanced nuclear transport of these proteins (Wang et al. 2021). All these changes enhance cellular efficiency under stress and are known to be crucial for adaptation to hypoxia (Wang et al. 2021).

**FIGURE 3:**
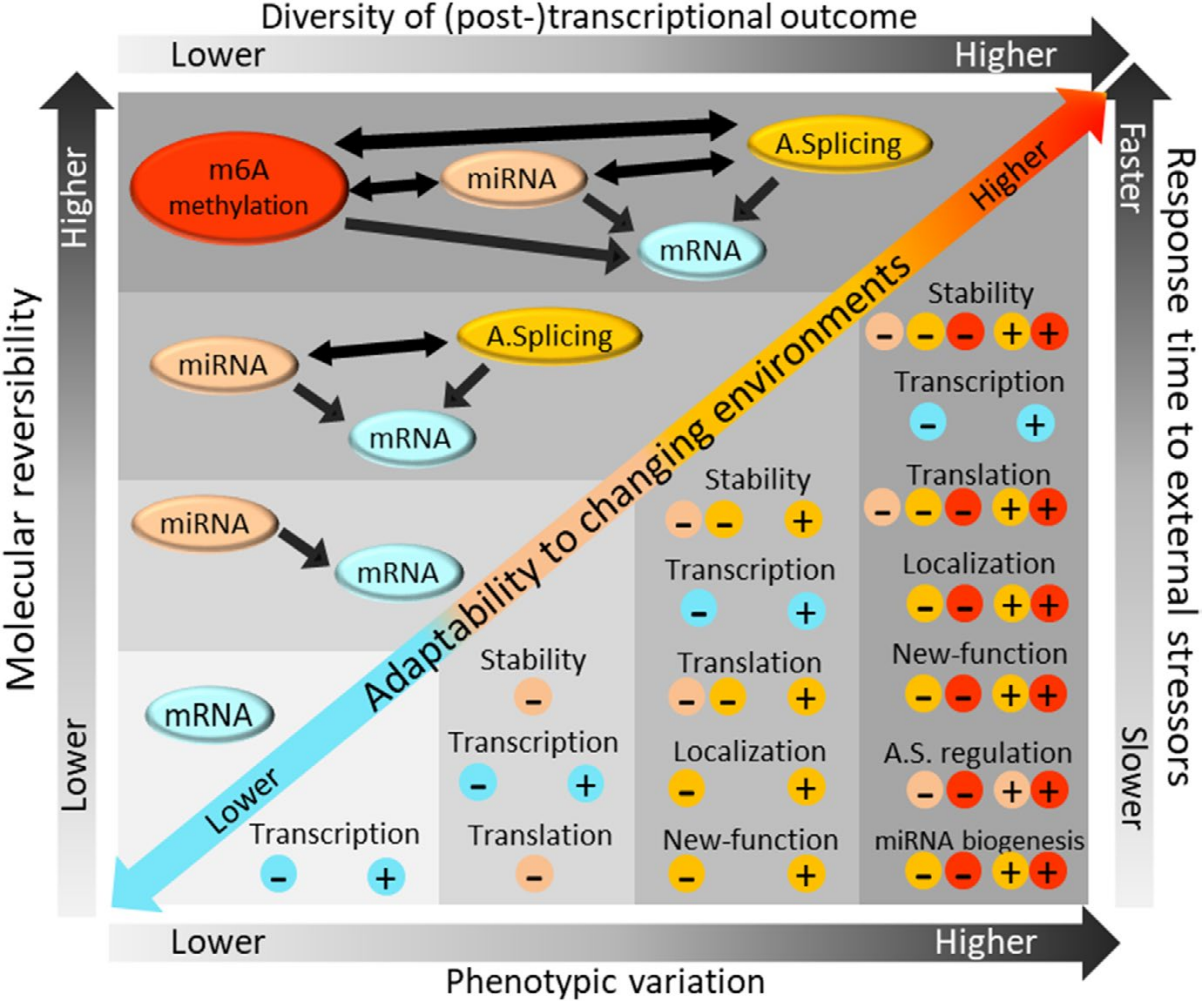
Potential enhancement of adaptability to changing environments through m^6^A RNA modification. In the higher compartment, black arrows indicate regulatory interactions between major (post‐) transcriptional processes. In the lower compartment, minus and plus signs, respectively, indicate inhibition/decrease and enhancement/increase in the specified RNA‐related biological processes, and their colour codes imply on the underlying (post‐) transcriptional mechanism.

In essence, m^6^A RNA methylation acts as a versatile conduit, enabling organisms to respond to environmental cues by orchestrating post‐transcriptional responses through miRNA regulation and alternative splicing. This can ultimately enhance their adaptive evolutionary potential (see a summary Figure 3). Thus, m^6^A RNA methylation is emerging as the missing piece of the puzzle in rapid and reversible environmental adjustment. The adaptive flexibility that m^6^A RNA methylation can potentially confer makes it a key player in the evolutionary toolbox.

Finally, it is important to note that while m^6^A RNA modifications significantly enhance the diversity of molecular responses to environmental changes—either through direct regulation of mRNA fates or by influencing other mechanisms (such as miRNA and splicing)—a substantial portion of these responses may not necessarily be advantageous and could even have negative impacts at individual or population level. Therefore, it is essential to conduct future studies of organisms in their natural environments to explore the conditions under which various m^6^A RNA modifications can result in adaptive, neutral or even maladaptive outcomes. A relevant example of this can be the emerging role of m^6^A RNA modification in complex developmental and physiological functions of brain, which is suggested as a contributing factor in both adaptive or maladaptive behaviours (M. Engel and Chen 2018). As fine‐tuning of transcriptional and translation is central to normal brain function, regulation of m^6^A RNA methylation has tremendous potentials in enhancing diversity of behaviours by introducing another layer of potentially regulated and stimulus‐adaptive gene expression control beyond simple changes in gene transcription (e.g., via affecting RNA maturation, stability, location and availability to protein translation) (M. Engel and Chen 2018).

## 6 m^6^A Interactions With microRNA and AS Mediating Plasticity

Phenotypic plasticity, the ability of organisms to adjust their traits and behaviours in response to changing environmental conditions, is considered a key component of adaptation (Fox et al. 2019). However, it is not always adaptive and can sometimes lead to maladaptive outcomes. In this nuanced role, plasticity contributes to how species respond to dynamic ecological landscapes and gene transcription changes are central in this process (Schneider & Meyer, 2017), as the ability to modulate gene expression in response to environmental cues allows organisms to adjust their molecular responses. These transcriptional changes can potentially lead to the development of new traits or the adjustment of existing ones, providing a mechanism for potential adaptation. Thus, while plasticity driven by gene transcription alterations offers a way for organisms to adapt, its role in evolutionary success is complex and depends on the specific environmental context and the nature of the changes (Stern et al. 2007). As plasticity is not inherently adaptive, distinguishing between adaptive and non‐adaptive gene regulatory outcomes of plastic responses presents a significant challenge. To effectively discern these effects, in‐depth molecular studies in natural or near‐natural settings are crucial. Longitudinal field studies that track genetic and RNA methylation changes, combined with controlled experiments, can shed light on the adaptive significance of plasticity.

Post‐transcriptional regulatory mechanisms, notably miRNA regulation and alternative splicing, are of paramount importance in the context of adaptive plasticity (Steward et al. 2022). These mechanisms serve as versatile and dynamic tools that organisms employ to swiftly respond to changing environmental conditions and fine‐tune their gene expression profiles (Steward et al. 2022). Together, these post‐transcriptional mechanisms play a critical role in adaptive plasticity, ensuring that organisms can thrive in an ever‐changing world. m^6^A RNA methylation is increasingly recognised as a potentially pivotal player in the realm of adaptive plasticity due to its unique attributes (Livneh et al. 2020; Scarrow, Wang, and Sun 2021). Unlike other post‐transcriptional regulatory mechanisms, m^6^A modifications offer an unparalleled combination of speed and reversibility in responding to environmental changes. These modifications can be rapidly installed, removed or reinstated on RNA transcripts, providing organisms with a highly flexible and dynamic means of fine‐tuning gene expression in response to shifting ecological conditions. Despite its potential significance, it is surprising that the role of m^6^A RNA methylation in adaptive plasticity remains relatively unexplored and underappreciated. The traditional focus in the field of post‐transcriptional regulation has predominantly revolved around miRNAs and alternative splicing, overlooking the swift and adjustable nature of m^6^A modifications. As a result, a critical knowledge gap persists, hindering our understanding of how this molecular mechanism may contribute to an organism's ability to rapidly adjust its gene expression profile in response to environmental cues.

In recent years, an increasing number of studies have highlighted the potential role of m^6^A RNA modification in phenotypic plasticity across various organisms. These studies draw attention to how distinct developmental and physiological processes influenced by m^6^A modifications may potentially hold adaptive value in diverse environments. For instance, in neural systems, m^6^A modifications enable rapid behavioural adjustments by modulating synaptic plasticity and memory functions. Studies show that under stress or memory‐inducing activities, the decrease in FTO levels leads to increased m^6^A levels, which facilitate the translation of memory‐promoting transcripts via the m^6^A reader YTHDF1. This dynamic regulation acts as a synaptic ‘activation switch’, exemplifying the impact of m^6^A RNA modifications on neuronal plasticity in response to external stimuli (Engel et al. 2018; Livneh et al. 2020; Walters et al. 2017). Other examples in animals include the distinct worker and queen castes of the honeybee (Apis mellifera) serve as models for studying the genomic mechanisms of phenotypic plasticity. This role is attributed to their flexible alternative splicing of hundreds of mRNAs for developmental genes during the various differentiation stages of the two castes (He et al. 2022). Interestingly, this flexibility is also linked to extensive m^6^A RNA methylation of these transcripts, with worker larvae exhibiting more hypermethylated m^6^A marks than queen larvae. Further functional investigations have shown that suppressing m^6^A RNA methylation in worker larvae induces queen caste features (Wang et al. 2021). These changes depend on the differential, developmentally dependent expression of various components of m^6^A RNA modifications, such as readers and writers (though not erasers). In Atlantic salmon, the differential expression of m^6^A modification markers, the m^6^A eraser *alkbh5* and the reader *ythdf2.2*, has been linked to variations in sex and pubertal timing during maturation which is an important life‐history trait (Ahi, Verta et al. 2024) Ahi, Frapin et al. 2024. Malaria parasites (Plasmodium spp.) also exhibit translational plasticity regulated by m^6^A, with the distribution of epitranscriptome marks across developmental stages playing a pivotal role in their adaptability to varying environmental conditions. This process is governed by the interaction of m^6^A readers YTH1 and YTH2, which dictate mRNA fate, underscoring the importance of m^6^A in the adaptability of these parasites (Govindaraju and Rajavelu 2024). In plants, m^6^A RNA modifications influence developmental timing and immune responses. For example, the m^6^A reader MhYTP2 in apple plants enhances resistance to fungal infections by promoting the translation of antioxidant genes and degrading susceptibility gene mRNAs, showing how m^6^A can drive immune adaptation (Guo et al. 2022). Similarly, in *Arabidopsis*, variability in the activity of the m^6^A eraser ALKBH10B affects flowering times, demonstrating the potential role of m^6^A in developmental adaptability (Duan et al. 2018). These modifications also extend to the regulation of gene expression in response to viral infections, as seen with ALKBH9B's role in modulating RNA virus infectivity (Martínez‐Pérez et al. 2017). In polyploid plants, m^6^A RNA methylation has been suggested as a potential key mechanism underlying their enhanced adaptive plasticity (Scarrow, Wang, and Sun 2021). This is likely due to increased m^6^A‐dependent silencing of deleterious chimeric‐transposable element transcripts and synergistic coordination of gene expression regulation and responses (Scarrow, Wang, and Sun 2021). These examples across diverse taxa highlight the various roles of m^6^A RNA modifications on phenotypic plasticity, showcasing its potential to mediate adaptive responses to environmental changes through a variety of biological processes. However, plasticity is not always adaptive and can sometimes lead to maladaptive outcomes. Understanding how frequently m^6^A contributes to negative plastic responses is an avenue for future research.

In future research, delving deeper into the potential roles of m^6^A RNA methylation in phenotypic plasticity could unveil novel insights into the mechanisms driving adaptive plasticity and species diversification. In essence, m^6^A modifications may represent a hidden key to understanding how organisms adjust to their ever‐changing environments, despite their surprising neglect in previous research. These modifications are dynamic and reversible, affecting RNA stability, localisation and translation efficiency, which enables cells to swiftly adjust their gene expression in response to environmental cues. Such acclimation is vital for phenotypic plasticity; for example, rapid changes in mRNA stability in response to stress can alter protein levels, aiding organisms in adapting to new conditions. m^6^A modifications also play significant roles in developmental processes and stress responses across various organisms. In plants, they are crucial for timing flowering and adapting to temperature changes (Duan et al. 2018; Scarrow, Wang, and Sun 2021), while in mammals, they influence immune responses and neuronal functions (Nainar et al. 2016; Shulman and Stern‐Ginossar 2020). Additionally, m^6^A impacts major signalling pathways involved in both phenotypic plasticity and environmental sensing, such as the Wnt and Hippo pathways (Budnik and Salinas 2011; Posfai et al. 2017; Qiao et al. 2021; Tabnak et al. 2023), and it interacts with epigenetic mechanisms like DNA methylation and histone modifications (Y. Zhao et al. 2021). This interplay may help integrate diverse signalling pathways and environmental factors, leading to coordinated responses that directly influence phenotypes. Understanding these interactions enhances our comprehension of the complex regulatory networks that drive phenotypic plasticity.

## 7 Embracing a 3D View in Future Studies of Post‐Transcriptional Mechanisms in Adaptive Evolution

Until recently, most studies focusing on post‐transcriptional regulation in the context of adaptive evolutionary research have largely adhered to a two‐dimensional perspective. Within this framework, the primary focus has been on elucidating interactions between established post‐transcriptional mechanisms like miRNA‐mediated regulation and alternative splicing with mRNA targets. This paradigm has significantly advanced our understanding of how these mechanisms shape gene expression and contribute to adaptive plasticity. However, it is essential to recognise that these post‐transcriptional mechanisms not only exhibit functional interferences and crosstalk among themselves (Shomron and Levy 2009) but also engage in reciprocal crosstalk with another crucial player—m^6^A RNA methylation (T. Chen et al. 2015).

It is interesting to note that both miRNAs and alternative splicing reciprocally regulate or interfere in m^6^A RNA methylation as well. For instance, some miRNAs have been found to directly target m^6^A writers, such as METTL3, inhibiting their expression (Cui et al. 2020). This reduces the deposition of m^6^A marks on target transcripts, altering their post‐transcriptional regulation. Conversely, certain miRNAs can target m^6^A erasers like FTO (Yang et al. 2022) or ALKBH5 (Xue et al. 2021), potentially modulating the removal of m^6^A modifications. This dual regulatory relationship between miRNAs and m^6^A RNA methylation illustrates the interplay between post‐transcriptional mechanisms, with miRNAs not only responding to m^6^A modifications but also influencing their deposition and removal (Mei et al. 2023). In addition, alternative splicing can indirectly impact m^6^A RNA methylation by affecting the expression of m^6^A writers, erasers or readers (Zhu et al. 2023). The splicing factor RBM15, for instance, can influence the abundance of METTL3, thereby modulating m^6^A levels on target transcripts (Wei et al. 2021). This bidirectional crosstalk between alternative splicing and m^6^A methylation reveals a complex regulatory relationship, where changes in splicing patterns can result in alterations in m^6^A modifications and vice versa.

Various methods have been developed over the past decades to assess m^6^A RNA modification spatially and temporally at both low and high throughput. This has facilitated the integration of data on m^6^A RNA dynamics with conventional analyses of mRNA, miRNA and alternative splicing within the same biological samples. Among the most commonly used methods for assessing m^6^A methylation in RNA are sequencing‐based technologies such as m^6^A‐CLIP, MeRIP‐seq (or m^6^A‐seq), m^6^A‐LAIC‐seq and the recently developed eTAM‐seq, each offering distinct advantages and disadvantages. In the context of ecological studies, MeRIP‐seq is advantageous as it is the most established and widely used technique, providing a broad overview of m^6^A modification across the transcriptome (Meyer et al. 2012) (Figure 2A). This makes MeRIP‐seq suitable for exploratory studies that aim to characterise the m^6^A landscape under various environmental conditions. However, it is not accurate enough when it comes to identifying the exact site of m^6^A methylation (McIntyre et al. 2020). On the contrary, m^6^A‐CLIP is more favoured in studies designed to pinpoint the exact locations of m^6^A modifications due to its superior specificity and resolution (Ke et al. 2015). However, it includes an extra laboratory step of UV cross‐linking, which makes it technically more challenging, especially when using very precious and sensitive biological samples. m^6^A‐LAIC‐seq may be the best method for further analyses at the splice‐variant/isoform level since it offers a quantitative approach to measure the abundance of m^6^A modifications in different RNA isoforms (Molinie et al. 2016). This is particularly useful in assessing how alternative splicing might affect m^6^A modification patterns or vice versa. However, this method also comes with certain setbacks; for example, it is technically more complex than MeRIP‐seq, requiring careful handling and analysis to differentiate among isoforms (Owens, Zhang, and Liu 2021). It requires more sequencing depth and computational resources and provides less resolution of the m^6^A site since it does not offer the single‐nucleotide resolution that techniques like m^6^A‐CLIP provide. Notably, all these methods also have the capacity to be adjusted to target only m^6^A modification in miRNAs, as has already been developed for m^6^A‐CLIP (miCLIP) (Owens, Zhang, and Liu 2021). However, due to the technical complexity of these methods, their usefulness in ecological studies requires further scrutiny. Recent modifications of these methods, such as MeRIP‐seq, are underway to make them low cost and low input, making them amenable to ecological and evolutionary research (Xia et al. 2024). One of the major recent advances is a technique called eTAM‐seq, which not only provides both high resolution and transcriptome‐wide assessment capacities but also works with very low amounts of input (Y. L. Xiao et al. 2023). This may make eTAM‐seq a valuable tool for ecological investigations. It even opens new avenues to study m^6^A modification using environmental RNA (eRNA), which is rapidly gaining interest and offers enormous potential for ecological health biomonitoring (Hechler et al. 2023; Yates, Derry, and Cristescu 2021).

Non‐sequencing‐based techniques such as quantitative mass spectrometry, CRISPR/Cas9‐based methods, MeRIP‐qPCR and total m^6^A RNA quantification offer unique advantages in ecological and environmental studies. Quantitative mass spectrometry provides precise quantification of m^6^A levels and directly measures RNA modifications (Glasner et al. 2017), which are crucial for linking specific RNA methylation changes to phenotypic adaptations under various environmental conditions. This method is particularly useful for rapid assessment of methylation changes and is applicable to a broad range of organisms, including those without well‐characterised genomes. CRISPR/Cas9 has enabled targeted manipulation of m^6^A modification machinery, allowing researchers to explore causal relationships between these modifications and adaptive responses, and to engineer organisms with modified m^6^A landscapes to study their impact on ecological fitness and adaptation strategies (Liu et al. 2019). Interestingly, the engineered m^6^A erasers by fusing CRISPR‐Cas9 with ALKBH5 or FTO are already achieved which enables site‐specific demethylation of RNAs. The development of such powerful programmable m^6^A RNA editing technologies promise ground‐breaking discoveries in various fields including molecular ecology by facilitating targeted and reversible manipulations of non‐model organisms in natural or semi‐natural settings and mechanistic understanding of epitranscriptomic responses to environmental challenges (Liu et al. 2019). An example of such methods is recently designed through a light‐inducible RNA m^6^A editing system with CRISPR‐Cas13 to direct specific m^6^A editing (Zhao et al. 2020). Strikingly, when combined with a nanoparticle film, this technique can work under near‐infrared wavelengths that penetrate tissues, offering a promising way to remotely control RNA editing (Zhao et al. 2020). The potential of this method is illustrated by studies of m^6^A‐mediated adaptive responses in virus‐host interactions (Bayoumi and Munir 2021). Finally, for more specific, low‐budget and low‐throughput but quick assessments of RNA modifications, particularly for preliminary studies before deploying more complex sequencing‐based technologies, MeRIP‐qPCR and total m^6^A RNA quantification—a commercially available ELISA‐based technique that quantifies the total m^6^A level in a given RNA sample—can also be the methods of choice (Ensinck et al. 2023; Harcourt et al. 2013). These approaches provide direct insights into the biological roles of specific m^6^A sites and support a comprehensive understanding of m^6^A's impact on gene expression regulation.

Taken together, to gain a comprehensive understanding of (mal)adaptive molecular mechanisms in various organisms, it is crucial for future studies to transition from the traditional two‐dimensional view to a three‐dimensional regulatory network perspective. Embracing this holistic framework will allow researchers to uncover the intricate interplay between miRNA regulation, alternative splicing and m^6^A RNA methylation. It will provide insights into how these post‐transcriptional mechanisms collectively shape gene expression profiles in response to environmental change and natural selection. By considering this three‐dimensional network view, we can better elucidate the complex and dynamic processes that underlie adaptive evolution, ultimately advancing our understanding of how organisms adjust and thrive in diverse environments.

## Author Contributions

E.P.A. and P.S. conceived the study; E.P.A. and P.S. drafted the manuscript, with E.P.A. having the main contribution, and both authors approved the final version of manuscript.

## Conflicts of Interest

The authors declare no conflicts of interest.

## Data Availability

Not applicable. No data were used in this manuscript.
